# Decentralized Cycle-Free Game-Theoretic Adaptive Traffic Signal Control: Model Enhancement and Testing on Isolated Signalized Intersections

**DOI:** 10.3390/s25206339

**Published:** 2025-10-14

**Authors:** Amr K. Shafik, Hesham A. Rakha

**Affiliations:** Charles E. Via, Jr. Department of Civil and Environmental Engineering, Virginia Tech, Blacksburg, VA 24061, USA; ashafik@vt.edu

**Keywords:** game theory, Nash bargaining, traffic signal control, adaptive signal control, signal timing, cycle length optimization, intelligent transportation systems

## Abstract

This research enhances and evaluates the performance of a Decentralized Nash Bargaining (DNB) adaptive traffic signal controller that operates a flexible National Electrical Manufacturers Association (NEMA) phasing and timing scheme responding dynamically to fluctuating traffic demands. The DNB controller is enhanced to (1) use traffic density estimates instead of queues to optimize signal timings; (2) to consider the eight-phase two-ring NEMA controller configuration within the game-theoretic approach; and (3) to consider dynamically adaptable control time steps. The enhanced DNB controller is benchmarked against (1) a fixed-time traffic signal control using the state-of-practice Webster’s method and an emerging Laguna-Du-Rakha (LDR) method for computing the optimum cycle length; (2) a state-of-the-practice actuated traffic signal control; and (3) a state-of-the-art reinforcement learning (RL) traffic signal controller presented in the literature. The controller is tested on two isolated signalized intersections, demonstrating enhanced overall intersection performance compared to the baseline pretimed and actuated controllers at various demand levels, and offers better performance than a previously developed RL controller. Specifically, the DNB controller results in a decrease in the average vehicle delay and queue size by up to 54% and 63%, respectively, compared to Webster’s state-of-the-practice pretimed control. Unlike the RL controller, the DNB controller requires no pre-training while adapting to fluctuating traffic conditions, thereby providing a flexible framework for reducing traffic congestion at signalized intersections. As such, this research contributes to the development of smarter and more responsive urban traffic control systems.

## 1. Introduction

Traffic signal control (TSC) strategies are classified into three most commonly used types: pretimed, actuated, and adaptive [1]. Pretimed TSC is derived offline based on the knowledge of the historical traffic flow and turning movements for each of the intersection approaches. The traffic signal is optimized to minimize the average vehicle delay and maximize the intersection capacity utilization. A phasing scheme is also designed to achieve the optimization goal, where each phase is given a split according to its relative flow-to-saturation ratio compared to other phases. The optimal cycle length is obtained from a non-linear total delay function such as that derived by Webster and Cobbe [2], which is currently the practice. However, some research efforts, such as the work of Calle-Laguna et al. [3], showed that the cycle length provided by Webster’s formula is overestimated. In addition, they provided an alternative formula to compute the optimal cycle length (the Laguna-Du-Rakha [LDR] formula). The authors demonstrated that the LDR formula provides a shorter cycle length that outperforms Webster’s formulation in terms of intersection delay and fuel consumption.

A major limitation of pretimed TSC is its insensitivity to the dynamic nature of traffic flow, which can fluctuate significantly in short periods of time. As such, adaptive TSC is introduced to re-evaluate traffic conditions in real time and optimize signal timings. The adaptive traffic signal controller uses data obtained from traffic sensors, such as traffic cameras or inductive loop detectors [4].

Building on this, recent studies have explored more advanced methods for both data collection and system optimization within adaptive traffic management. For instance, Akopov et al. [5] present a novel approach using a parallel genetic algorithm to enhance the maneuverability and safety of a multi-agent fuzzy transportation system. Their research goes beyond simple signal timing, focusing on optimizing the overall road network layout to improve vehicle flow and increase system efficiency in real time. In another study, Nam et al. [6] address the data acquisition challenge by proposing a deep learning model to estimate traffic density. Their approach uses a Long Short-Term Memory artificial neural network that processes data from connected and autonomous vehicles, providing a modern, cost-effective alternative to traditional traffic sensors. Together, these examples highlight the shift towards using sophisticated computational intelligence and advanced data sources to build smarter, more responsive adaptive transportation systems.

In addition, actuated TSC is also used to mitigate the effect of traffic stochasticity on the optimal signal plan through vehicle actuations [1], where it allows phases to be skipped or ended if there is no traffic demand. However, the design of actuated traffic signals is based on an equivalent optimal pretimed timing plan [7], where the maximum green interval is obtained using the pretimed green durations multiplied by a factor ranging from 1.25 to 1.50 [8]. As such, the utilized cycle length that is optimized for a certain demand level is insensitive to real-time fluctuations in traffic demand. Furthermore, research efforts have proposed other types of TSC strategies, such as the use of neural networks for real-time optimization of TSC [9], and the implementation of reinforcement learning within adaptive TSC [10].

### 1.1. Related Work

Recent research efforts have utilized the concepts of game theory for the traffic signal optimization problem. The problem is perceived as a game of multiple rational participants who coordinate interactively and cooperate to achieve the maximum benefit, where the players’ decisions represent rational individual behavior. Game theory is considered an important tool in many fields, such as communication, economics, business, politics, psychology, and evolutionary biology. In the transportation field, game theory has been used in multiple areas, such as route choice modeling, traffic management, logistics, transportation systems analysis, as well as traffic control [11,12,13,14,15].

Research efforts have utilized game theory in the traffic domain, such as in the work of Bazzan, which utilized techniques of evolutionary game theory for traffic signal coordination [16]. Alvarez and Poznyak applied game theory to the traffic control problem. Their method was applied to an isolated intersection, where the Nash bargaining (NB) controller outperformed adaptive TSC in terms of queue length minimization [17]. Linglong et al. also showed that the game-theoretic TSC algorithm improved the control performance compared to pretimed TSC, where the framework was applied as a two-player game [15]. Bui et al. also developed a two-player game framework to optimize traffic signal timings. The methodology is based on two game models: the Cournot Model and the Stackelberg Model [18]. However, this method is constrained by a fixed cycle length that is calculated in advance. In addition, this method is only applicable to two-phase traffic signals.

Guo and Harmati compared Nash and Stackelberg equilibrium cooperative strategies to pretimed TSC [19]. Each player’s cost function was computed by dividing the queue length by the approach queue capacity. The cooperative strategies showed superior performance in terms of the vehicle queues and throughput compared to pretimed control. Guo and Harmati [20] also compared the game-theoretical strategy to single-agent reinforcement learning in TSC. The game-theoretical strategy showed a 6.02% improvement over the Q-learning strategy.

Abdelghafar and Rakha developed a decentralized NB (DNB) TSC that provides a flexible phasing scheme as well as phasing splits that adapt to changing demand levels. The system was tested in a simulation environment of a downtown Los Angeles network [21,22]. The study utilized the approach queue length to evaluate the payoff function for each player. (The full theoretical analysis and proof of convexity when applying the DNB methodology with the queue length method are provided in these articles). A significant performance improvement was reported, with up to a 64% reduction in vehicle delay. However, the study did not account for the intergreen time associated with each phase switch, which resulted in overestimated benefits. It is also noted that using queue length to evaluate the payoff function may overlook approaches with higher traffic density. Some of these shortcomings were addressed in Shafik and Rakha [23], in particular, the consideration of the intergreen times in the optimization and evaluation of the DNB controller. However, the study did not consider the National Electrical Manufacturers Association (NEMA) eight-phase controller and only considered the queue length in the objective function.

### 1.2. Study Contribution

This study advances the state-of-the-art in decentralized game-theoretic traffic signal control by addressing several gaps that have limited the practical deployment of earlier NB formulations, and then benchmarks it against state-of-the-practice fixed-time and actuated TSC. The specific contributions are as follows:

1. Integration with full NEMA eight-phase control: Unlike most prior game-theoretic controllers, which were limited to simplified two-phase or four-phase configurations, we implement the controller within the widely used NEMA eight-phase framework. This extension is critical for real-world applicability, as it allows the method to directly interface with existing traffic controller architectures in the US.
2. Explicit consideration of phase transition penalties: We incorporate yellow and all-red clearance intervals into the payoff evaluation process. This addition corrects a common limitation in earlier studies, which ignored intergreen times and consequently overstated potential benefits.
3. Density-based payoff evaluation: We redefine the bargaining utility function using the approach of traffic density rather than queue length. This methodological enhancement captures lane utilization more effectively, ensuring that approaches with high but dispersed demand are not undervalued, which is an issue that has affected previous NB implementations.
4. Adaptive evaluation horizon and minimum green enforcement: We introduce a dynamic control horizon that adjusts to the current queue discharge time across all phases. This ensures that each green interval is long enough to clear existing queues and meet driver expectancy, avoiding unrealistic or erratic phase changes while maintaining flexibility.
5. Comprehensive benchmarking: We benchmark the enhanced DNB controller not only against conventional pretimed (Webster and Laguna-Du-Rakha) and actuated controller strategies but also against a reinforcement learning-based controller reported in the literature. The results demonstrate that the DNB controller achieves significant improvements without the need for pre-training, highlighting its scalability and transferability across intersections.

These contributions move decentralized game-theoretic traffic signal control beyond conceptual demonstrations by extending the NB framework to the full NEMA eight-phase scheme, incorporating intergreen times and phase transition penalties into payoff evaluations, and replacing queue-based with density-based payoffs to capture approach utilization more effectively. These elements have not been incorporated together in previous studies, most of which relied on simplified two-phase models, ignored transition penalties, or evaluated payoffs only in terms of queues. Each step requires nontrivial adaptation and has a tangible effect on controller performance, addressing implementation fidelity, operational realism, and benchmarking rigor. The outcome is a controller that is both theoretically grounded and practically deployable in modern traffic management systems.

## 2. Traffic Signal Control Strategy

### 2.1. Overview of the DNB Algorithm

NB, which is a fundamental concept in cooperative game theory, is utilized in this study to develop a decentralized TSC algorithm. The developed DNB approach addresses the traffic signal optimization problem as a multi-player game, where each signal phase represents a rational player in the game. All players coordinate and cooperate to make an optimal decision that is agreed to by all parties, where each mutually agreeable decision involves a payoff for, or a penalty on, the players. The optimal NB solution guarantees that no alternative outcome can improve one player’s position without worsening the position of other players. As such, the optimal decision maximizes the product of the players’ payoff from their disagreement points, which is a key feature of NB.

The decentralized control terminology refers to a scenario in which the control strategy operates independently at each intersection. In contrast, a centralized strategy coordinates between traffic signals and imposes constraints on the NB decision by enforcing a specific offset and cycle length to maintain traffic signal coordination.

The enhanced DNB algorithm is implemented on the NEMA eight-phase phasing scheme, which provides a more dynamic phasing sequence. The DNB algorithm with variable phase durations ensures the optimal decision is taken by properly addressing the requirements of each player in the game-theoretic framework. This decision is made based on the evaluation of the expected total payoff to be received when giving a certain phase a green indication. In this process, each applicable scenario is evaluated, and the benefits or penalties are calculated for all players, resulting in a total payoff for all the available decision options.

The DNB controller does not depend on predefined or learned traffic patterns. Instead, it evaluates all possible phase scenarios in real time and selects the one with the highest utility, which is the one with the greatest overall benefit. This allows the controller to adapt dynamically to the varying traffic conditions on the intersection approaches, as long as the intersection remains undersaturated. In cases of oversaturation, where the optimization problem becomes infeasible, the system operates on a pretimed fallback control plan.

### 2.2. The DNB Players

In this problem, the game strategy is defined as identifying the player who receives the green indication, while other players receive red indications. Each player in the game represents a combination of non-conflicting phases that receive a green indication simultaneously. As such, the game players are defined as pairs of all possible combinations of non-conflicting phases, which are eight players representing the eight non-conflicting phase combinations in the NEMA phasing scheme [24].

### 2.3. The Disagreement Point

The disagreement point is defined as the threshold that defines the minimum acceptable utility level for player *j*, irrespective of the decision to be taken. If the utility ($u_{ij}$) in a given scenario falls below this point, the bargaining process becomes invalid with no guarantee for optimality.

In an undersaturated system, where demand does not exceed the capacity, bargaining scenarios are designed to ensure mutually agreeable outcomes, avoiding states of excessive queueing or unserved demand. This implies that the maximum achievable player utility ($u_{ij}$) for any player should always be less than or equal to the disagreement point. As such, each of the available decision options must satisfy this condition. This represents a benchmark for the minimum payoff that each player is willing to achieve.

In this problem, the disagreement point is represented by the maximum number of vehicles that can be stored for the lane groups associated with each player before one lane group spills back to other lanes or other upstream links, creating gridlock. The disagreement point is represented by $d_{j}$, which is calculated using the storage length of all the lanes that carry traffic that are discharged in the phases controlled by player *j*, as shown in Equation (1), where *P* is the phases included in player *j*, $L_{n}$ is the length of the lane *n*, *N* is the lanes included in phase *p*, and $k_{jam}$ is the lane jam density.

Please note that the maximum utility for any player is less than or equal to the disagreement point value, and a player’s utility cannot be greater than the disagreement point. Otherwise, this means that the disagreement point has already occurred, and the current process is invalid. As such, the maximum player’s utility, which corresponds to the minimum payoff, is always lower than the value of the disagreement point.

(1) $$d_{j}=\sum_{p}^{P}\sum_{n}^{N}L_{n}\times k_{jam}$$

### 2.4. The Payoff Evaluation Process

The process of payoff evaluation determines the payoff and penalties corresponding to each player at each of the available decisions, prior to decision-making. The payoff evaluation is based on the utility function $U_{i}$. The utility value of each player *j* for each decision *i* is represented by $u_{i,j}$. The player utility is computed for each player based on the approach density, where the number of vehicles corresponding to a phase leverages the utility value of this phase. The payoff value $U_{i}$ is calculated as shown in Equation (2) below, where the total utility value of the decision scenario *i* is the multiplication of the difference between the queue capacity and the utility corresponding to that player.

(2) $$U_{i}=\prod_{j=1}^{J}d_{j}-u_{i,j}$$

Table 1 illustrates an example of a *J*-player game-theoretic framework, where the utility is calculated at the available *I* game decision scenarios, where each player has a utility function $u_{i,j}$ for each scenario. The total payoff $U_{i}$ is calculated for each decision scenario using Equation (2). As such, the decision is taken based on the highest payoff value $U_{i}$, where the problem is constrained to prevent each player’s utility from reaching the disagreement point. The problem formulation can be written as shown in Equation (3a,b), where the set *S* represents the utility space.

(3a) $$\begin{array}{cc} \; & \max\;U_{i} \end{array}$$

(3b) $$\begin{array}{ccc} \; & s.t.\;u_{i,j}\in S,\;u_{i,j}\leq d_{j} & \end{array}$$

Equation (3b) shows that the maximum utility for any player is less than or equal to the disagreement point value, as was described earlier.

### 2.5. Traffic Density Prediction

The payoff utility function is calculated based on a future prediction of the traffic density of each approach for each potential decision. First, the information about the current utility value is obtained by assuming a fully connected traffic network, where every object in the simulation, such as vehicles, signals, and loop detectors, is assumed to be connected and sharing full information through vehicle-to-everything (V2X) communication, or using traffic cameras directed at each of the intersection approaches. As such, the spatio-temporal state of each vehicle in the network is known.

Second, the payoff of each prospective decision is estimated by predicting the future density on each approach as a result of each decision to be taken. The average traffic flow measured by loop detectors in the last 30 s on each approach is used to estimate the traffic density, where the loop detectors are positioned directly upstream of the disagreement point. This prediction is performed over a horizon period Δt, which also represents the DNB decision update period, where the DNB algorithm re-evaluates the current signal status versus the other scenarios, and decides whether to extend the current signal status or to switch to another player.

The traffic density computation formula (utility value) utilizes an input-output model to calculate the future traffic density (Equation (4)). When evaluating a certain phase that receives a green indication during the next Δt, the lost time is calculated by subtracting $t_{\mathrm{lost}}$ from the implementation period Δt, which takes into consideration the intergreen time (yellow and red clearance times) of the current phase as well as the start loss time for the next phase. In addition, when the phase is evaluated for receiving a red light while it is currently green, the lost time at each phase is considered in the density estimation. Please note that the start-up lost time and the end clearance lost time are computed based on the current status of the phase with respect to its next status. The consideration of the lost time at each prospective switching scenario penalizes the frequent phase-switching behavior, as the more frequent switches lead to increased overall lost time.

(4) $$u_{t+\Delta t}=\left\{\begin{array}{cc} u_{t}+q_{\mathrm{in}}\Delta t-q_{\mathrm{out}}\left(\Delta t-t_{\mathrm{lost}}\right), & \mathrm{at}\;\mathrm{green}\;\mathrm{phases}.\\ u_{t}+q_{\mathrm{in}}\Delta t-q_{\mathrm{out}}t_{\mathrm{eg}}, & \mathrm{at}\;\mathrm{red}\;\mathrm{phases}. \end{array}\right.$$

where $u_{t}$ is the current traffic density, $q_{in}$ is the average traffic flow for the previous 30 s obtained from the loop detector, and $q_{out}$ is the average discharge rate.

### 2.6. The Solution Procedure

The enhanced DNB algorithm conducts an evaluation checkpoint at each time interval Δt. All permissible phasing scenarios are evaluated at each checkpoint to determine whether to extend the current phases or switch to other phases. Once a decision is made using the utility function, the duration of the optimal phase is set to the queue discharge time corresponding to that phase.

**Figure 1:**
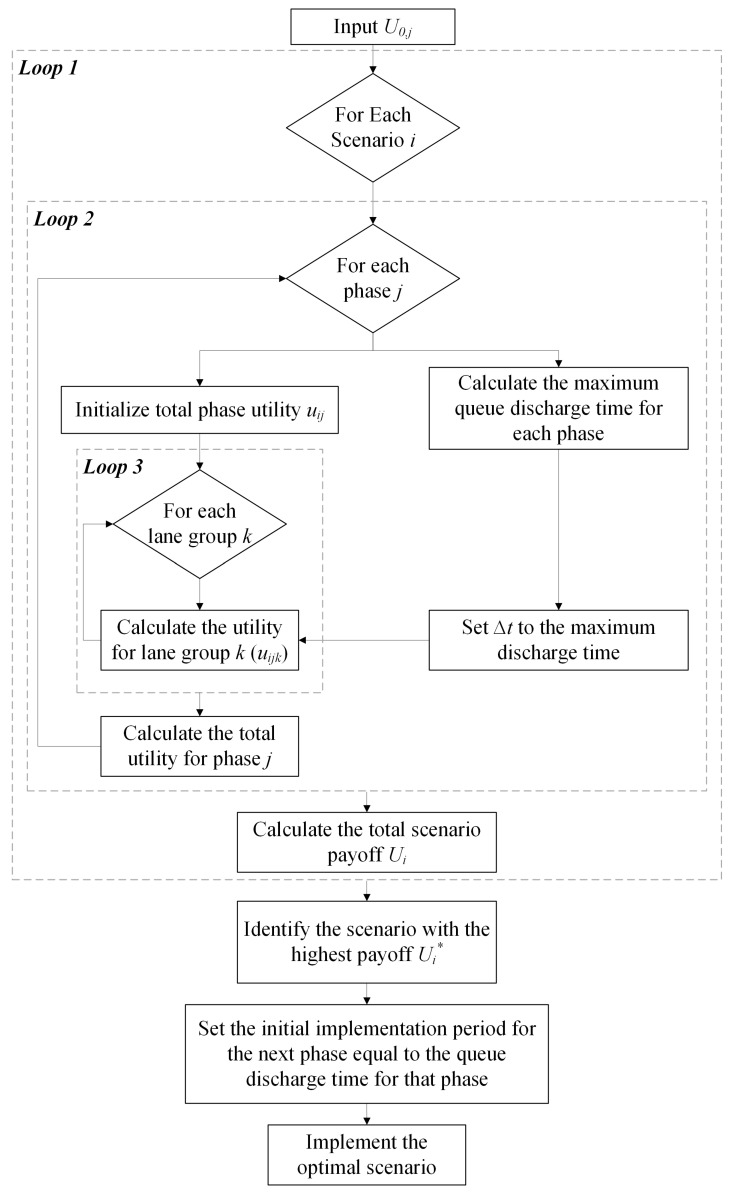
The DNB procedure framework.

At the end of the specified green duration, the DNB controller re-evaluates all scenarios to decide whether to extend the green time of the current phase or to switch to other phases. Algorithm 1 as well as Figure 1 outline the DNB solution procedure, which is executed at each evaluation period Δt.
**Algorithm 1** The DNB Algorithm**Input:**$\;u_{0,j}$: the current utility value at time $t_{0}$ for phase *j*.**for** each scenario *i* **do**   **for** each phase *j* **do**     Initialize total phase utility $u_{i,j}=0$     Calculate the queue discharge time for each phase.     Set Δt to the maximum discharge time.     **for** each lane group *k* in phase *j* **do**        Calculate lane group utility $u_{i,j,k}$ at $t_{0}+\Delta t$.        Update total phase utility $u_{i,j}+=u_{i,j,k}$     **end for**     Calculate total phase utility $u_{i,j}$ at $t_{0}+\Delta t$.   **end for**   Calculate total scenario payoff $U_{i}=\prod_{j}d_{j}-u_{i,j}$ at $t_{0}+\Delta t$.**end for**Determine the optimal scenario with the highest payoff $U_{i}$.Set the implementation period of the next phase to the queue discharge time of that phase.Implement the optimal scenario.

### 2.7. The DNB Evaluation Horizon

This approach ensures a minimum green time that clears the current queued vehicles in the subject phase, providing enough time for the drivers to react to the start of the green interval and meet driver expectancy.

The Δt, representing the evaluation horizon, is dynamically calculated at each checkpoint based on the current queueing status of each phase. Specifically, Δt is set to the maximum discharge time of the currently queued vehicles across all phases. This approach ensures that the minimum green time allocated to the critical phase is enough to clear the currently queued vehicles. This approach also ensures a dynamically estimated evaluation time, which is adjusted by the system in real time to be suitable for the current traffic demand level. In addition, this gives drivers sufficient time to react to the start of the green interval, meeting driver expectancy, as noted at the beginning of this section.

The queue discharge time is estimated using Equation (5) below, where the estimated time includes the start loss time ($t_{l}$) plus the actual queue discharge time, which is calculated using the discharge saturation flow rate, $q_{c}$, and the number of vehicles detected stopping at the stop bar $N_{q}$. Note also that the phase duration is lower-bounded (taken to be 10 s in this study) to meet driver expectancy at low demands and upper-bounded by a maximum green time to avoid excessively long phases at high demands. The maximum green time is determined by multiplying the optimal pretimed green duration by a factor of 1.5.

(5) $$t_{discharge}=t_{l}+N_{q}/q_{c}$$

Finally, a maximum red interval is also applied to minor approaches (taken to be 150 s). The red indication timer begins as soon as a vehicle is detected on the phase receiving a red indication. This mechanism helps prevent excessive delays at minor approaches, particularly when traffic demand is low.

## 3. The DNB Algorithm Experimental Setup

In this section, the enhanced DNB algorithm is evaluated using real traffic data from two isolated four-legged intersections: one located in downtown Toronto, Canada, and another in Orlando, Florida. The two intersections are modeled using a microscopic simulation software developed in the Python (3.13.5) environment [25]. The simulation software incorporates underlying traffic flow models as follows:

- The vehicle dynamics model for light-duty vehicles developed by Rakha et al. [26,27] is used to model vehicle motion. Maximum acceleration is determined by the maximum tractive force and instantaneous resisting forces, with the tractive force calculated from the vehicle’s maximum power. Resisting forces include rolling, aerodynamic, and grade resistance forces. These calculations are done at every time step (Δt).
  (6) $$\begin{array}{cc} a_{\max}^{\mathrm{DYN}} & =\frac{F_{\max}-R}{m} \end{array}$$
  (7) $$\begin{array}{cc} F_{\max} & =\min\left[3600\eta_{d}\beta \frac{P_{\max}}{v},M_{\mathrm{ta}}g\mu \right] \end{array}$$
  (8) $$\begin{array}{cc} R & =\frac{\rho }{25.91}C_{d}C_{h}A_{f}v^{2}+mg\frac{c_{r0}}{1000}\left(c_{r1}v+c_{r2}\right)+mgG \end{array}$$
- The Van Aerde steady-state car-following and traffic stream model [28], which is a single-regime model that combines the Greenshields and Pipes functional forms, is used to simulate the vehicle’s steady-state car-following behavior. The speed-density relationship is modeled using Equations (9)–(12).
  (9) $$\begin{array}{cc} k & =\frac{1}{c_{1}+\frac{c_{2}}{u_{f}-u}+c_{3}u} \end{array}$$
  (10) $$\begin{array}{cc} c_{1} & =\frac{u_{f}\left(2u_{c}-u_{f}\right)}{k_{j}u_{c}^{2}} \end{array}$$
  (11) $$\begin{array}{cc} c_{2} & =\frac{u_{f}{\left(u_{f}-u_{c}\right)}^{2}}{k_{j}u_{c}^{2}} \end{array}$$
  (12) $$\begin{array}{cc} c_{3} & =\frac{1}{q_{c}}+\frac{u_{f}}{k_{j}u_{c}^{2}} \end{array}$$
- The Fadhloun–Rakkha car-following model [29] is used to simulate human-driven vehicles, incorporating vehicle dynamics, steady-state car-following behavior, and collision avoidance strategies to maintain safe following distances. The model includes acceleration and collision avoidance regions, where throttle and deceleration levels are adjusted based on the current speed, spacing, and the lead vehicle’s behavior. Vehicle acceleration is expressed as a proportion of the maximum allowable acceleration (Equation (6)) using the throttle level ($f_{p}$). This model governs the dynamics of vehicles, ensuring collision avoidance by calculating the required deceleration using a vehicle kinematics model.
  (13) $$\begin{array}{cc} X_{n} & =\frac{s_{n}^{\mathrm{VA}}}{s_{n}}\cdot \frac{v_{n}}{v_{n}^{\mathrm{VA}}} \end{array}$$
  (14) $$\begin{array}{cc} f_{p} & =e^{-aX_{n}}{\left(1-X_{n}^{b}e^{b(1-X_{n})}\right)}^{d} \end{array}$$
  (15) $$\begin{array}{cc} \mathrm{CA} & =\min\left(d_{\mathrm{req}},\frac{d_{\mathrm{req}}^{2}}{d_{\mathrm{des}}+gG}\right) \end{array}$$
  (16) $$\begin{array}{cc} d_{\mathrm{req}} & =-1\times \left(\frac{v_{n}^{2}-v_{n-1}^{2}+\sqrt{{\left(v_{n}^{2}-v_{n-1}^{2}\right)}^{2}}}{4(s_{n}-s_{j})}\right) \end{array}$$
  (17) $$\begin{array}{cc} a_{n} & =\left\{\begin{array}{cc} f_{p}\times a_{\max}^{\mathrm{DYN}} & \forall X_{n}<1\\ \mathrm{CA} & \forall X_{n}\geq 1 \end{array}\right. \end{array}$$

Table 2 shows the description of the equation notations. Detailed descriptions of the case studies and benchmarks are provided in the following subsections.

### 3.1. Case Studies

#### 3.1.1. Case Study 1

The first case study incorporates a four-legged signalized intersection located in a downtown congested area in Toronto, Canada (the intersection of Front St. and Bay St.). This particular case study is chosen to benchmark the DNB results against the results of previous case studies that used the same intersection, such as El-Tantawy et al. [10].

**Figure 2:**
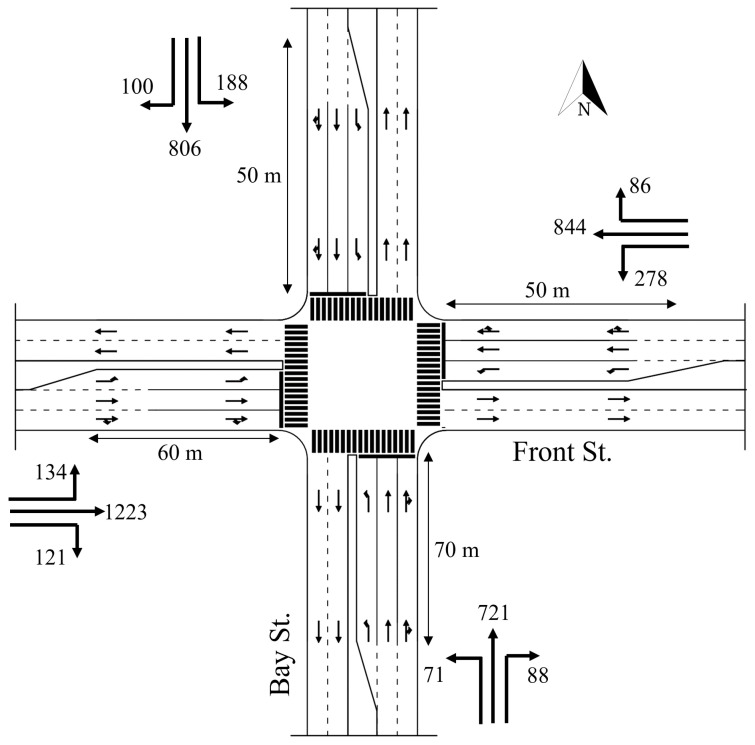
Case Study 1 Intersection layout and traffic demand in vehicles/hour (Front St. and Bay St., Toronto).

**Figure 3:**
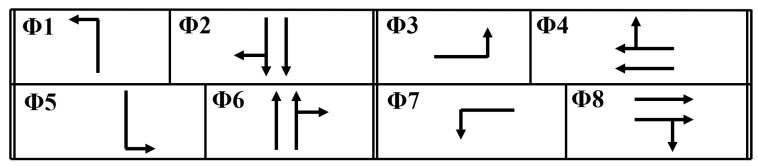
Toronto intersection phasing diagram.

The intersection features four approaches, each with an exclusive left-turn pocket lane. Figure 2 shows the 2005 intersection configuration and traffic demand during the PM peak hour, which represents the worst-case scenario at this intersection. The intersection data are obtained from El-Tantawy et al. [10]. Figure 3 shows the 8-phase intersection scheme considered in this study. The simulation model is calibrated so that the baseline results are consistent with the observed conditions and the base model as reported in the literature [10]. Furthermore, the demand input is modeled based on exponentially distributed arrivals, mimicking the random nature of vehicle arrivals. As such, the model can simulate the variability and randomness in traffic demand.

#### 3.1.2. Case Study 2

**Figure 4:**
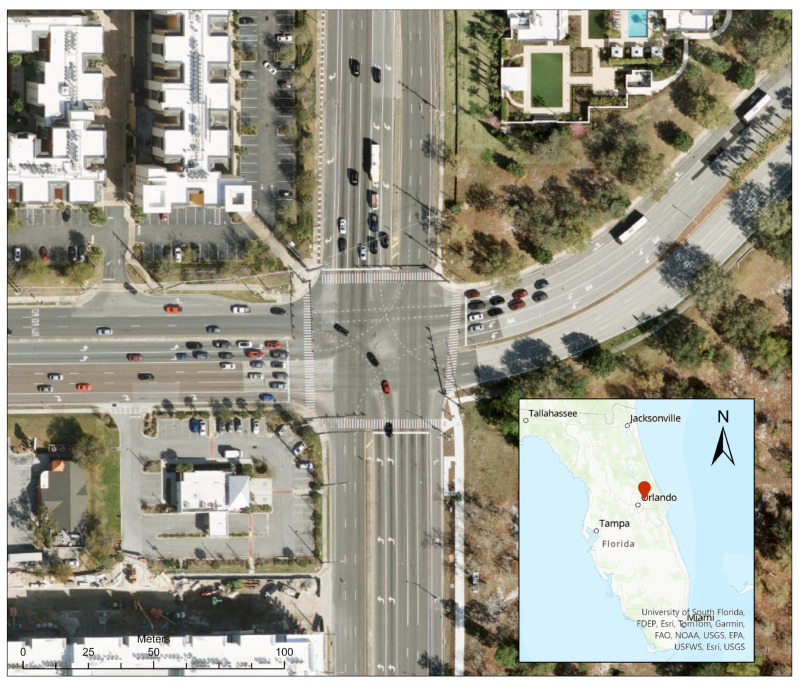
Case Study 2 Four-legged signalized intersection at Alafaya Trail and University Boulevard in Orlando, Florida.

**Figure 5:**
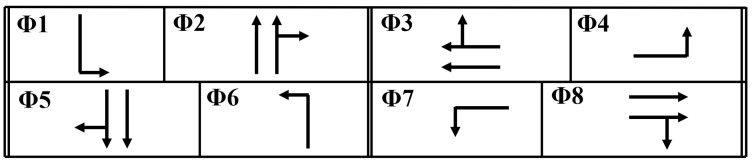
Orlando intersection phasing diagram.

This intersection represents real-world trajectory data sourced from a drone-based dataset [30]. This dataset is collected at a four-legged signalized intersection situated at the intersection of Alafaya Trail and University Boulevard in Orlando, Florida. This location, illustrated in Figure 4, serves as a second case study for a sensitivity analysis to assess the effectiveness of the DNB traffic signal controller at various traffic demand levels. The choice of this dataset provides a realistic and dynamic representation of urban traffic scenarios, showing the algorithm’s performance under real-world conditions of actual traffic patterns and oscillations. In addition, using this dataset ensures that the evaluation process is performed for typical conditions, enhancing the credibility of the system’s performance assessment. The phasing scheme utilized in this study is the same as observed from the field, as shown in Figure 5. Furthermore, various demand levels for this intersection are simulated to evaluate the algorithm’s performance in critical conditions.

### 3.2. Model Calibration and Validation

In order to ensure that the simulation software provides a valid abstraction of reality, the model parameters are calibrated using empirical trajectory data. Subsequently, the model is validated by comparing estimated delays and queues to those derived from empirical data. This validation process ensures the validity of the simulation software and its usefulness in the process of modeling and testing the proposed signal control system.

#### 3.2.1. Calibration of Model Parameters

The driver behavior parameters (*a*, *b*, and *d*) were calibrated using trajectory data obtained from a field experiment conducted at the Virginia Smart Roads test facility at the Virginia Tech Transportation Institute [31]. In addition, the model’s behavior was closely monitored to ensure it produced realistic and reasonable driving patterns. Acceleration profiles from 250 trajectories involving 15 drivers were utilized to calibrate the Fadhloun–Rakha car-following model parameters: *a*, *b*, and *d*. The calibration was performed by minimizing the error in travel time and fuel consumption between the simulated and actual speed profiles for all trajectories. This process yielded model parameters of 0.74, 1.00, and 0.10 for the parameters *a*, *b*, and *d*, respectively.

The fundamental diagram traffic stream parameters, including free-flow speed, jam density, saturation flow rate, and speed-at-capacity for case study 1, were obtained from El-Tantawy et al. [10]. For the second case study, the parameters were derived from the drone-based trajectory data using the following procedure:

- The free-flow speed is the speed at or below which 85% of the vehicles are observed to travel under free-flow conditions, which is observed in this case to be 54.8 mph (88.3 km/h), where the posted speed limit is 45 mph.
- The saturation flow rate is computed using the average vehicle flow rate when discharging from a queue.
- The speed-at-capacity is obtained by measuring the average vehicle speeds when discharging from a queue.
- The jam density is obtained by measuring the traffic stream density when vehicles are queued during the red signal indication.

The parameters of both case studies are shown in Table 3.

#### 3.2.2. Model Validation

The validation process for the simulation models of the case studies is performed by comparing measures of performance, including average vehicle delay and average number of queued vehicles. Regarding case study 1, the simulation model for the base case scenario yielded an average delay of 48.5 s/veh, where the reported average delay by El-Tantawy et al. in this case was 48.9 s/veh [10].

**Figure 6:**
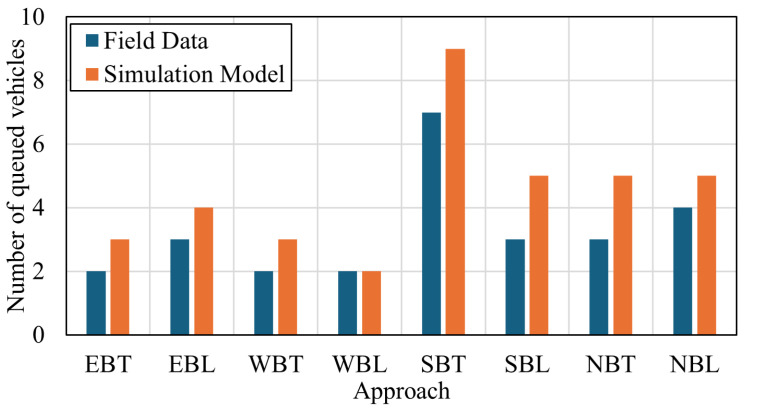
Average number of queued vehicles on each approach.

For the second case study, given that drone-based trajectory data are available, the validation procedure is performed using the average delay and the average number of vehicles in queue on each approach. The field trajectory data yielded an average delay of 25.96 s/veh, while the simulation model reported a delay of 25.62 s/veh, resulting in a 1.1% error. The average number of queued vehicles is illustrated in Figure 6, where the root mean square error is 1.4 vehicles.

### 3.3. Benchmarks

The performance of the DNB algorithm is compared with the corresponding optimal pretimed and actuated control strategies based on intersection performance metrics, such as vehicle delay and queue length. Two methods are used to determine the optimal cycle length for the pretimed control: Webster’s method [2] and the LDR method [3], The cycle length formulas for both methods are shown in Equation (18), where *L* is the total lost time and *Y* is the sum of critical phase flow ratios. The baseline control method considered is the pretimed control using Webster’s cycle length.

(18) $$C=\left\{\begin{array}{cc} \frac{1.5L+5}{1-Y}, & \text{Webster’s}\;\text{Formula},\\ 39.3\ln\left(\frac{L}{1-Y}\right)-75.7, & \text{LDR’s}\;\text{Formula}. \end{array}\right.$$

The fully actuated signal control system is also chosen as a benchmark since it is known for its efficiency in adapting to fluctuating demand. It considers vehicle detections for all traffic movements, which allows the controller to adjust phase lengths and skip phases as needed, resulting in efficient green time allocation on a cycle-by-cycle basis. It also enables phase skipping if no vehicles are detected for a given phase. The actuated signal controller provides the minimum green time to each phase whenever a vehicle detection triggers a service call. The green signal is extended by the passage time if additional vehicles are detected with headways shorter than the minimum allowable headway. Otherwise, the green indication is terminated if no vehicles are detected in the current phase and conflicting demand is present. Additionally, the green indication for a phase is terminated if it exceeds the maximum allowable green time for that phase.

The fully actuated signal control design is performed based on the recommended Federal Highway Administration guidelines, where the minimum green durations are set as 5 s for left turns and 15 s for through movements [32]. The maximum green interval is obtained using the factored pretimed green durations, where the optimum timings are multiplied by a factor of 1.25 [8]. The passage time is calculated as 3.1 s using a detector length of 35 ft, vehicle length of 20 ft, and the minimum allowable headway of 4 s.

## 4. Results and Analysis

This section presents the simulation results of the enhanced DNB traffic signal controller. Performance metrics of the DNB controller are extracted and compared with the optimal pretimed and actuated traffic signal control strategies. The following subsections present the detailed results and analysis of the system performance for each of the case studies.

### 4.1. Results of Case Study 1

#### 4.1.1. Optimal Cycle Length Computation

In this intersection, the cycle length is calculated using both Webster’s and LDR’s formulas based on the road capacity parameters, such as the saturation flow rate and the speed limit, which are obtained from El-Tantawy et al. [10]. For the base case condition, Webster’s optimal cycle length is calculated to be 170 s, and the LDR’s optimal cycle length is calculated to be 100 s.

#### 4.1.2. Vehicle Delay

Simulation results show that the DNB controller significantly outperforms both Webster’s and LDR’s pretimed plans in terms of reducing average delay. Table 4 presents the average delay results for each of the signal control strategies. The table shows that a delay reduction of up to 37.9% is achieved by the DNB controller compared to Webster’s pretimed plan. The LDR pretimed and actuated plans also demonstrate better performance compared to the state-of-practice Webster’s method, with a reduction in average delay by 25.8% and 32.0%, respectively. The results show that the DNB controller outperforms the optimal pretimed and actuated strategies, indicating a significant improvement in signal control efficiency.

Additionally, the DNB controller demonstrates more delay reductions compared to the RL approach. In the same case study, El-Tantawy et al. [10] reported average delays of 39.5 s for the pretimed controller and 33.33 s for the adaptive controller using the RL algorithm. Given that the baseline performance in our study closely matches El-Tantawy et al.’s, it can be concluded that the DNB algorithm outperforms the RL approach. Furthermore, a key limitation of the RL algorithm is its need for pre-training on an intersection, which limits its transferability to other settings. In contrast, the DNB controller does not require pre-training and can be readily applied to different intersections regardless of their specific characteristics.

#### 4.1.3. Fuel Consumption

In terms of fuel consumption, the average fuel consumption reduction rates by the DNB controller and LDR’s pretimed plan are 10.9% and 11.0%, respectively, compared to Webster’s pretimed plan, as shown in Table 5. The table shows that the enhanced DNB controller produces fuel consumption savings comparable to the performance of the actuated controller, which is the state-of-the-practice.

#### 4.1.4. Queue Size

**Figure 7:**
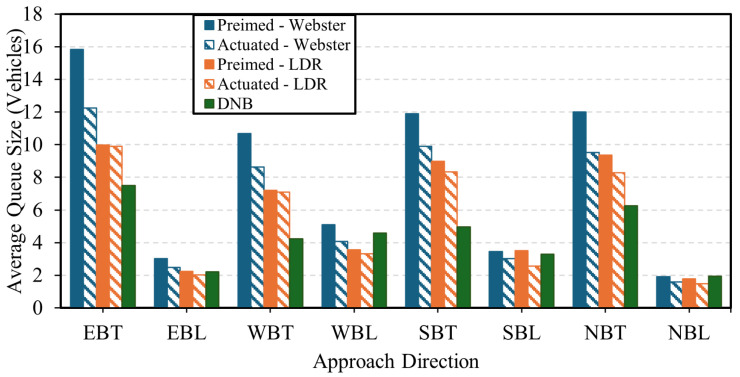
Average queue length for each approach and movement Combination-Case Study 1.

In terms of average vehicle queues, the DNB controller showed improved performance over the pretimed and actuated control strategies on major approaches. Figure 7 presents the average queue length for the pretimed control, the actuated control, and the DNB controller. The figure shows that the pretimed and actuated plans using the LDR formula demonstrate better performance compared to Webster’s method, producing shorter queue lengths, on average. Additionally, the DNB controller results in lower average queues compared to both pretimed and actuated control strategies, resulting in a reduction in the average queue length across all approaches by 45% compared to the baseline pretimed plan, while the LDR-actuated plan results in an average queue reduction of 33%.

#### 4.1.5. Variation of the Cycle Length and Phase Splits

**Figure 8:**
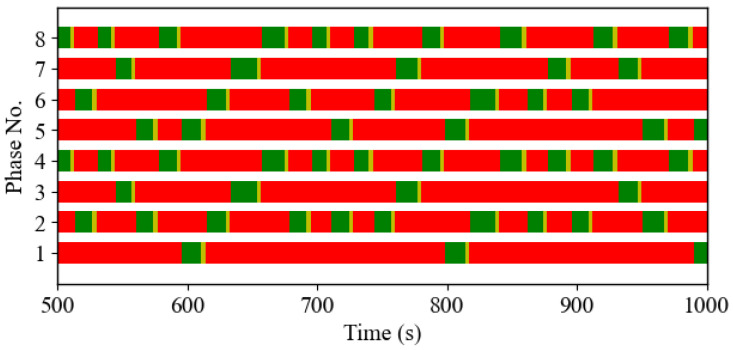
Temporal variation in the traffic signal indications resulting from the DNB controller-Case Study 1.

Figure 8 shows the switching pattern for the DNB controller between 500 and 1000 s. This figure demonstrates that the DNB controller provides dynamic phase-switching and timing. This increased flexibility in both the phase-switching sequence and the allocated green time durations for each phase allows the DNB controller to achieve more efficient traffic management, coping with traffic fluctuations in real time.

**Figure 9:**
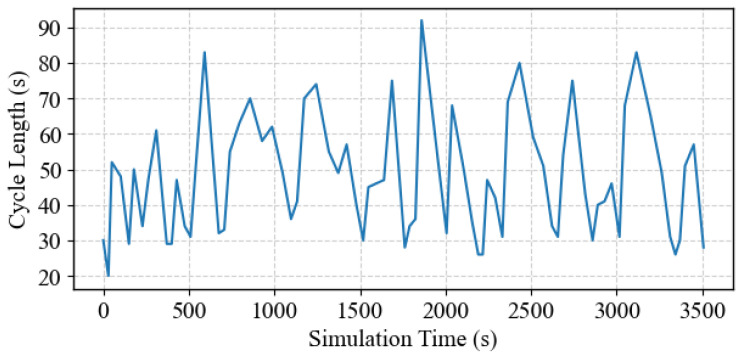
Cycle length variation during the simulation-Case Study 1.

**Figure 10:**
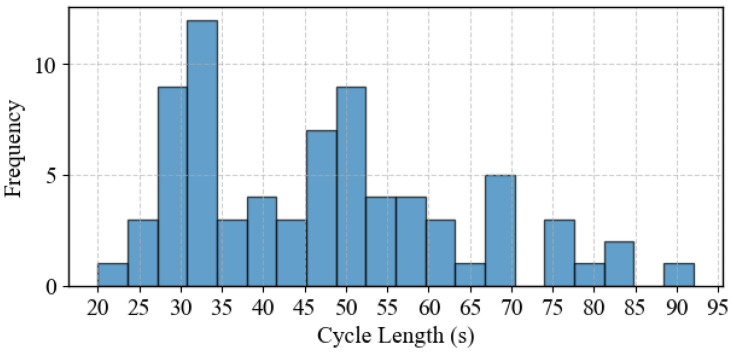
Cycle length distribution during the simulation-Case Study 1.

Figure 9 illustrates the variation in the cycle length produced by the DNB controller, where the cycle length is calculated from the end of the critical phase (Phase No. 8 in this case). The figure shows that the cycle length fluctuates between approximately 20 s and 90 s. These fluctuations indicate that the DNB controller, while not explicitly optimizing the cycle length, generates a dynamically varying cycle length to address the demand needs. This adaptability helps optimize traffic flow and reduce traffic congestion on the intersection approaches. Furthermore, Figure 10 also displays the distribution of the cycle lengths for the entire simulation, where the cycle length of around 30 s occurs more frequently. This demonstrates the DNB controller’s flexibility in adjusting the cycle length dynamically, according to the real-time demand level, further demonstrating its ability to adjust the intersection operation in response to varying traffic conditions.

#### 4.1.6. Number of Vehicles in the System

**Figure 11:**
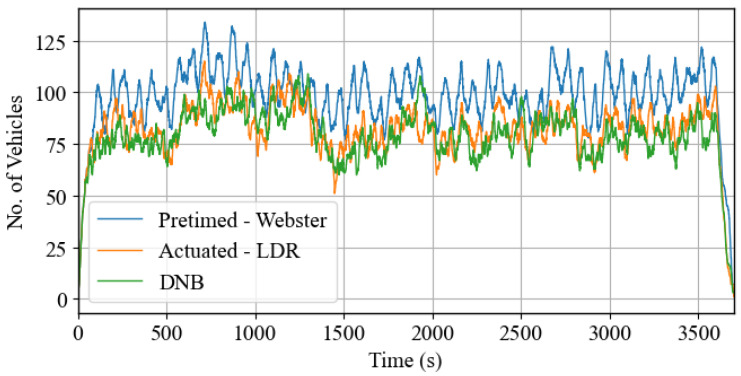
Number of vehicles concurrently on the network for the entire simulation-Case Study 1.

Figure 11 illustrates the number of vehicles in the system throughout the entire simulation period for three scenarios: the baseline Webster’s pretimed control, the LDR-actuated control, and the DNB controller. This figure provides a visual representation of how the total number of vehicles in the system fluctuates with each implemented signal control plan. In this comparison, Webster’s pretimed plan results in the highest number of vehicles present in the system. This suggests that Webster’s pretimed plan may not be the most efficient in terms of minimizing congestion and managing traffic flow. The LDR-actuated plan also results in a relatively higher number of vehicles, although not as many as Webster’s plan. On the other hand, the DNB controller generally results in a lower number of vehicles in the system throughout the simulation period. This indicates that the DNB controller is more effective in optimizing traffic flow and reducing congestion compared to the pretimed and actuated plans. The lower vehicle count associated with the DNB controller suggests that it may be better suited for dynamic traffic conditions, providing more responsive adjustments to traffic signal timings based on real-time traffic data.

### 4.2. Results of Case Study 2

#### 4.2.1. Optimal Cycle Length Computation

In this intersection, a sensitivity analysis is performed to compare the DNB controller with the optimal pretimed and actuated control strategies at various levels of traffic demand, where the sum of critical Y ratios (volume to saturation flow ratio) ranges from 0.05 to 0.9. The yellow and all-red clearance intervals of 4 and 1 s, the cycle lengths corresponding to each of the demand levels, are shown in Table 6 using both LDR’s and Wenbster’s formulas (Equation (18)).

The table shows that the LDR formulation provides a much shorter cycle length compared to Webster’s formulation at high demand levels. For example, Webster’s formula provides a 415-s cycle length at a Y ratio of 0.9, while the LDR’s formula provides an optimal cycle length of 140 s. Please note that the sum of flow ratios, as computed from trajectory data, is represented by the point Y = 0.5.

#### 4.2.2. Vehicle Delay

**Figure 12:**
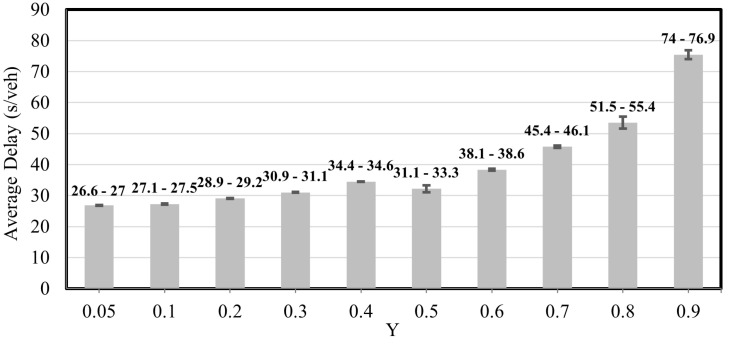
Average Delay Results with Margin of Error-Case Study 2.

Figure 12 illustrates the relationship between the demand level Y (the sum of critical y ratios) and the average delay experienced by vehicles. The bars include error bars, which indicate the range of variability across random seeds for each demand level. This shows that the reported delays are not fixed values but fall within specific confidence intervals, demonstrating the consistency and robustness of the proposed algorithm across multiple simulation runs.

**Figure 13:**
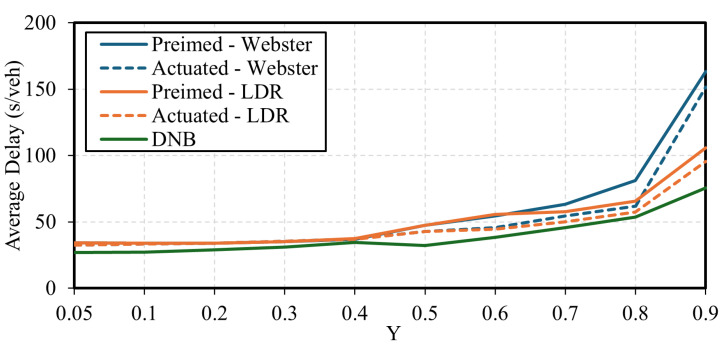
Average vehicle delay for the various control strategies-Case Study 2.

Figure 13 presents the average vehicle delay results for each demand level and control strategy. The results show that the LDR cycle length formula consistently outperforms Webster’s formula, providing lower delays at Y ratios greater than 0.5 under pretimed and actuated signal control. In addition, the figure shows that the DNB controller consistently provides lower average delays across all demand levels.

**Figure 14:**
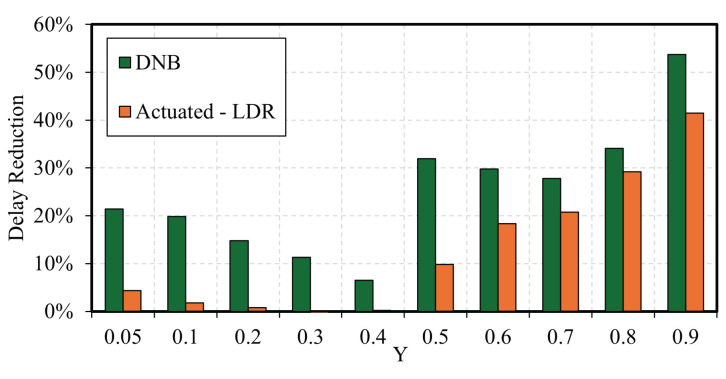
Delay reduction compared to the base pretimed control-Case Study 2.

Figure 14 shows a comparison of the average vehicle delay benefits for the best-performing control strategies: the DNB and the LDR-based actuated controller, where the baseline is the Webster-based pretimed control. The figure shows that the DNB algorithm outperforms the actuated control, where the delay benefits reach 54% compared to the optimal pretimed plan. The LDR-based actuated controller shows delay reduction benefits up to 41.5%.

To assess whether the top two performing systems (the DNB and the Actuated-LDR systems) differ significantly, an analysis of variance (ANOVA) test was conducted at each demand level using an alpha level of 0.05. Table 7 reports the *p*-values for the various demand levels (*Y* ratios). The results indicate that the differences are statistically significant in nearly all cases, with *p*-values below the 0.05 threshold across the tested levels. This shows that the observed differences are unlikely to be due to random variation and that the performance gap between the two systems is consistently significant across different demand levels.

To further evaluate performance, a one-way ANOVA was conducted to compare the DNB and LDR-based actuated control strategies, the best-performing methods. The null hypothesis is that the average delay of the two methods is equal. Simulations were run with 10 different random seeds, each lasting 1 h. The ANOVA revealed a statistically significant difference in average vehicle delay between the two strategies, across all demand levels (p<0.05). Post-hoc comparisons using Tukey’s Honestly Significant Difference (HSD) test showed that the DNB controller significantly outperforms the LDR-based actuated control for all demand levels. These findings indicate that the DNB controller delivers significantly better performance than the LDR-based control at various demand levels.

It is also observed that at low traffic demand levels, actuated signals can experience premature gap-outs due to the longer headways between vehicles. This occurs when the vehicle headway exceeds the minimum allowable headway set in the actuated control strategy, leading to premature termination of the green phase and switching to the next phase. This leads to control inefficiencies of the actuated traffic signal at medium demand levels compared to the pretimed optimal control, such as at Y = 0.3 and 0.4, as shown in Figure 14.

#### 4.2.3. Queue Size

**Figure 15:**
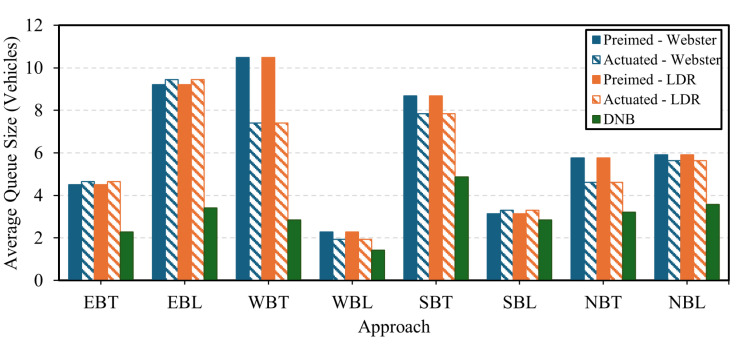
Average Intersection Queue Length Per Approach-Case Study 2.

**Figure 16:**
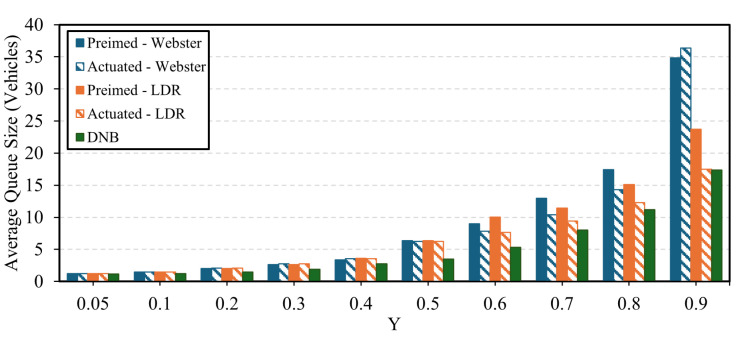
Average Intersection Queue Length as a function of the Sum of the Intersection Y Ratio-Case Study 2.

**Figure 17:**
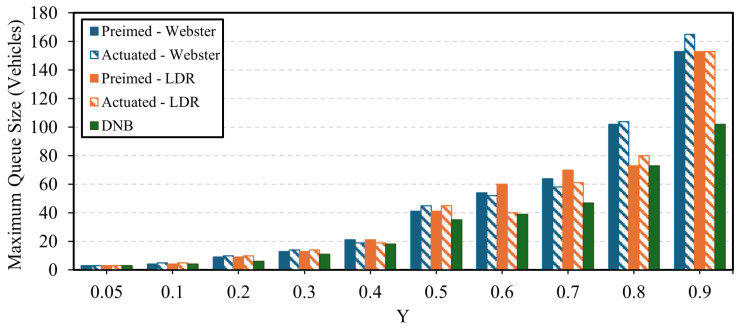
Maximum Intersection Queue Length as a function of the Sum of the Intersection Y Ratio-Case Study 2.

In terms of queue size results, the DNB controller showed significant results in terms of queue reduction compared to Webster’s, the state-of-the-practice, pretimed, and actuated signal control. Figure 15 shows the average queue size at the current demand level for each approach at the field-observed demand level. The figure shows that the LDR pretimed plan continues to outperform Webster’s plan. In addition, the DNB also reduces the overall average queue size by up to 63% compared to the LDR optimal pretimed plan. Figure 16 and Figure 17 show the average and maximum queue size, respectively, at each demand level. The DNB controller outperforms all other control strategies at various demand levels.

### 4.3. Discussion

The results presented in the previous sections highlight the effectiveness of the DNB controller in managing traffic signals under fluctuating traffic patterns. Compared to state-of-the-practice methods, the DNB controller significantly reduces average vehicle delay and queue sizes across various traffic demand levels. Additionally, the performance of the DNB in reducing the average delay outperforms the reported benefits of the RL algorithm for traffic signal control at the same demand level [10]. In addition, the DNB algorithm is more transferable to other intersections, unlike the RL method, which requires pre-training and is intersection-specific, and cannot be applied beyond the scope of the training data.

This is achieved by dynamically responding to real-time traffic conditions without the need for manual adjustments. The DNB controller adapts to current demand patterns and traffic densities within the intersection approaches. This is a key advantage of the DNB controller, which, unlike pretimed and actuated control strategies, is optimized for specific demand patterns and lacks real-time adaptability, whereas the DNB controller adjusts its timing and phase-switching in real time. This dynamic nature allows it to adapt in scenarios where traffic demand fluctuates frequently, maintaining optimal traffic movements without parameter recalibration.

Another consideration is the unexpected timing and switching patterns generated by the DNB controller, which may be unfamiliar to drivers. To enhance driver expectancy, introducing a minimum green time concept ensures that vehicles already queued can clear the intersection, aligning with the objective of minimum green times in actuated traffic signals [32].

In comparing traffic signal control strategies, it is important to note that the benefits of pretimed and actuated controls may be overestimated, as these plans are optimized in this analysis at each specific static demand pattern. However, in the field, these control strategies do not dynamically update their parameters in response to real-time traffic fluctuations, even in scenarios with full connectivity. This lack of adaptability contrasts with the DNB controller, which continuously optimizes signal timings based on live traffic conditions.

In terms of the computational cost of the proposed DNB controller, it is considered generally higher than actuated and fixed-time traffic signal control because of the processing of traffic data needed for the estimation and prediction of vehicle queues and traffic stream density. However, the DNB’s computation time is low, since the calculation of the utility function of all scenarios is not a complicated procedure; basically, it involves the multiplication of terms equal to the number of players. We quantified the computational time for the DNB controller in both case studies described in this article. The runtime for the DNB runs is recorded at each evaluation step for a simulation period of 1 h. The average run times are 0.05 and 0.06 s, which are repeated each evaluation time step for case studies 1 and 2, respectively. The variation in runtime reflects the variation in the intersection geometry, where more lanes result in more traffic, which requires more processing time. These results show that the runtime of the proposed system is still very minimal and suitable for real-time deployment.

The current DNB controller is primarily vehicle-focused and does not explicitly include pedestrians, cyclists, or transit modes as players in the game-theoretic framework; however, that is a promising direction for future research. It should be noted that the model can accommodate pedestrian needs by enforcing minimum green times that ensure enough time for crossing, with timing adjusted based on intersection geometry and local requirements. Alternatively, a dedicated pedestrian/bicycle phase can easily be incorporated by adding a player representing this phase. With regard to buses, transit priority can be addressed by adding more weight to the buses when computing the utility function or by dynamically changing the disagreement point to make a certain phase more favorable for buses.

It should be noted that the DNB traffic signal controller relies on real-time traffic state data, specifically vehicle density and queue length within the control region, to make optimal decisions. A key challenge in deploying the system is acquiring, estimating, and predicting these data. While full V2X capabilities would provide ideal input, low market penetration of connected vehicles remains a significant barrier. Alternatively, the system could integrate data from roadside sensors such as traffic cameras, loop detectors, and/or LIDAR; however, this approach may require substantial infrastructure investment.

Finally, it is also noted that this study is limited to two simulated isolated intersections, which were selected to serve as proof-of-concept case studies for evaluating the DNB controller across a range of traffic scenarios and demand levels. While this scope is sufficient for demonstrating the feasibility and potential benefits of the proposed approach, we recognize that further validation is necessary. Future work will focus on extending the evaluation to larger, more complex networks and pursuing field implementation studies to assess the controller’s performance under real-world conditions.

Despite the promising results, several limitations of this study should be acknowledged. First, the study is limited to two simulated isolated intersections. These case studies were chosen to provide a proof-of-concept evaluation of the DNB controller with various scenarios and demand levels. However, it should be noted that previous versions of the DNB controller were tested on an arterial in Blacksburg, VA, and on a downtown Los Angeles network comprised of 457 signalized intersections.

## 5. Conclusions and Future Work

The enhanced DNB controller is compared to optimal pretimed and actuated traffic signal control strategies, where the cycle length is computed using two methods: the state-of-practice Webster, as well as the state-of-the-art LDR formulations. The testing is carried out in a simulation environment at two isolated signalized intersections using field-observed traffic demands. The first case study is a highly congested isolated intersection that provides a benchmark comparison to machine learning controllers. The second case study is an isolated intersection where drone-based trajectory data are available. Multiple demand levels are simulated on the latter signalized intersection to test critical control scenarios.

In the first case study, the DNB controller produces a 38% reduction in vehicle delay relative to the Webster-derived fixed-time plan, which outperforms the delay savings of an RL controller. In the second case study, the DNB controller achieves benefits in average delay of up to 54%, exceeding the savings produced by replacing the Webster method with the LDR cycle length optimizer in pretimed and actuated control mode. The DNB controller also produces up to 45% and 63% savings in the average queue size for the two case studies, respectively, which in turn outperform pretimed and actuated control strategies. Unlike the RL controller, the DNB controller requires no pre-training, making it ideal for field implementations.

A key input to the DNB controller is the traffic stream density on the signal approaches and the saturation flow rates of the various traffic movements. Consequently, future research work includes integrating Kalman filters with the DNB controller to address incomplete and erroneous data in real-time traffic state estimation [33]. Furthermore, research is needed to integrate the DNB controller with the optimization of vehicle trajectories [34,35] to form an integrated traffic optimization system for urban networks. Future research directions include a deeper assessment of computational cost, scalability under large-scale deployments, and the long-term reliability of the controller in diverse traffic environments.

## Figures and Tables

**Table 1:** Game Decision Scenarios and Payoffs.

Scenario	Players				Total Payoff
	1	2	…	J	
Scenario 1	Green $u_{1,1}$	Red $u_{1,2}$	…	Red $u_{1,J}$	$U_{1}$
Scenario 2	Red $u_{2,1}$	Green $u_{2,2}$	…	Red $u_{2,J}$	$U_{2}$
…	…	…	…	…	…
Scenario I	Red $u_{I,1}$	Red $u_{I,2}$	…	Green $u_{I,J}$	$U_{I}$

**Table 2:** Notation Description.

Notation	Description
***Vehicle Dynamics***	
$f_{b}$	Throttle input
$\eta_{d}$	Driveline efficiency
β	Constant accounting for gear shift impacts
$M_{ta}$	Vehicle mass acting on the tractive axle
μ	Road friction or adhesion coefficient
ρ	Air density at sea level and 25 °C
$C_{d}$	Drag coefficient
$C_{h}$	Altitude correction factor
$A_{f}$	Vehicle frontal area
$c_{r0}$, $c_{r1}$, $c_{r2}$	Rolling resistance constants
***Van Aerde Traffic Stream Model***	
k,	Traffic density, and the jam density.
$u,u_{f},u_{c}$	Vehicle speed, the free-flow speed, and the speed at capacity.
$c_{1}$	Fixed distance headway constant
$c_{2}$	First variable headway constant
$c_{3}$	Second variable distance headway constant
***Car-Following Model***	
a,b,d	Car-following model parameters
$s_{n}^{\mathrm{VA}},s_{n},s_{j}$	Steady-state spacing, current vehicle spacing, and jam spacing, respectively
$v_{n}^{\mathrm{VA}},v_{n},v_{n-1}$	Steady-state speed, follower speed, and leader vehicle speed, respectively
$d_{req},d_{des}$	Kinematic (required) and desired deceleration levels, respectively

**Table 3:** Traffic stream parameters.

Parameter	Case Study 1	Case Study 2
Saturation Flow Rate (veh/h/ln)	1900	1800
Free-flow Speed (km/h)	40.0	88.3
Speed-at-Capacity (km/h)	25.0	40.0
Jam Density (veh/km)	160	114

**Table 4:** Average Delay Results.

Control Type		Average Delay (s/veh)	Improvement
Pretimed	Webster	48.5	-
	LDR	36.0	25.8%
Actuated	Webster	38.7	20.3%
	LDR	33.0	32.0%
DNB		30.1	37.9%

**Table 5:** Average Fuel Consumption Results-Case Study 1.

Control Type		Average FC (mL)	Improvement
Pretimed	Webster	77.2	-
	LDR	70.5	8.6%
Actuated	Webster	71.7	7.1%
	LDR	68.7	11.0%
DNB		68.8	10.9%

**Table 6:** The Optimal Cycle Length at Each Demand Level-Case Study 2.

Y	Cycle Length (s)	
	LDR	Webster
0.05	60	60
0.1	60	60
0.2	60	60
0.3	60	60
0.4	65	60
0.5 (Actual Demand)	75	75
0.6	85	105
0.7	100	140
0.8	115	205
0.9	140	415

**Table 7:** Comparison of mean values, standard deviations, and ANOVA *p*-values between DNB and Actuated-LDR across different demand levels (*Y* ratios).

Y Ratio	Mean		SD		*p*-Value
	DNB	Actuated-LDR	DNB	Actuated-LDR	
0.05	26.80	32.62	0.323	0.215	$2.37\times 10^{-20}$
0.10	27.28	33.42	0.331	0.305	$1.29\times 10^{-19}$
0.20	29.06	33.83	0.244	0.106	$9.30\times 10^{-22}$
0.30	31.03	35.31	0.169	0.089	$1.82\times 10^{-23}$
0.40	34.48	36.80	0.198	0.126	$4.04\times 10^{-17}$
0.50	32.20	42.69	0.607	0.021	$1.88\times 10^{-21}$
0.60	38.33	44.56	0.506	0.008	$7.82\times 10^{-19}$
0.70	45.75	50.21	0.757	0.028	$3.31\times 10^{-13}$
0.80	53.49	57.50	3.736	0.349	$3.34\times 10^{-3}$
0.90	75.47	95.50	2.759	11.247	$3.39\times 10^{-5}$

## Data Availability

Data are contained within the article.

## Abbreviations

The following abbreviations are used in this manuscript:

DNB	Decentralized Nash Bargaining
VOC	Volume-to-Capacity Ratio
NB	Nash Bargaining
NEMA	National Electrical Manufacturers Association
TSC	Traffic Signal Controller
